# Hypoxia‐driven paracrine osteopontin/integrin αvβ3 signaling promotes pancreatic cancer cell epithelial–mesenchymal transition and cancer stem cell‐like properties by modulating forkhead box protein M1

**DOI:** 10.1002/1878-0261.12399

**Published:** 2018-12-22

**Authors:** Junyu Cao, Jie Li, Liankang Sun, Tao Qin, Ying Xiao, Ke Chen, Weikun Qian, Wanxing Duan, Jianjun Lei, Jiguang Ma, Qingyong Ma, Liang Han

**Affiliations:** ^1^ Department of Hepatobiliary Surgery First Affiliated Hospital Xi'an Jiaotong University China; ^2^ Department of Anesthesiology First Affiliated Hospital Xi'an Jiaotong University China

**Keywords:** cancer stem cell‐like properties, epithelial–mesenchymal transition, hypoxia, pancreatic cancer, pancreatic stellate cells

## Abstract

Pancreatic stellate cells (PSCs), a key component of the tumor microenvironment, contribute to tumor invasion, metastasis, and chemoresistance. Osteopontin (OPN), a phosphorylated glycoprotein, is overexpressed in pancreatic cancer. However, OPN expression in PSCs and its potential roles in tumor–stroma interactions remain unclear. The present study first showed that OPN is highly expressed and secreted in activated PSCs driven by hypoxia, and this process is in a ROS‐dependent manner; in addition, OPN was shown to be involved in the PSC‐induced epithelial–mesenchymal transition (EMT) and cancer stem cell (CSC)‐like properties of pancreatic cancer cells (PCCs). Mechanistically, OPN from activated PSCs interacts with the transmembrane receptor integrin αvβ3 on PCCs to upregulate forkhead box protein M1 (FOXM1) expression and induce malignant phenotypes of PCCs. Moreover, the Akt and Erk pathways participate in OPN/integrin αvβ3 axis‐induced FOXM1 expression of PCCs. Our further analysis showed that OPN and FOXM1 are significantly upregulated in pancreatic cancer tissues and are associated with poor clinical outcome, indicating that OPN and FOXM1 might be considered as diagnostic and prognostic biomarkers for patients with pancreatic cancer. In conclusion, we show here for the first time that OPN promotes the EMT and CSC‐like properties of PCCs by activating the integrin αvβ3‐Akt/Erk‐FOXM1 cascade in a paracrine manner, suggesting that targeting the tumor microenvironment represents a promising therapeutic strategy in pancreatic cancer.

**Abbreviations**
α‐SMA: α‐smooth muscle
CSC: cancer stem cell
ECM: extracellular matrix
EMT: epithelial–mesenchymal transition
FOXM1: forkhead box protein M1
MMP: matrix metalloproteinase
NAC: N‐acetyl‐l‐cysteine
OPN: osteopontin
PCC: pancreatic cancer cell
PSC: pancreatic stellate cell
ROS: reactive oxygen species
TCGA: the Cancer Genome Atlas
TST: thiostrepton

## 1 Introduction

With a 5‐year survival rate less than 8% and more than 44 000 deaths per year, pancreatic cancer represents one of the most lethal human cancers and is the fourth leading cause of cancer‐related death in the United States (Siegel *et al*., 2018). The quality of life and prognosis of patients with pancreatic cancer are dismal due to the early invasion and metastasis, as well as the limited efficacy of therapeutic strategies (Ryan *et al*., 2014). Despite decades of efforts, pancreatic cancer remains a substantial fortress awaiting conquest.

Hypoxia is a characteristic feature of human solid tumors, including pancreatic cancer. Increasing evidence has demonstrated that hypoxia is strongly associated with enhanced tumor invasiveness, angiogenesis, and distant metastasis (Du *et al*., 2015; Li *et al*., 2017). In addition, our previous studies also showed that low oxygen tension could induce pancreatic cancer cell (PCC) invasion and epithelial–mesenchymal transition (EMT) (Cao *et al*., 2016; Lei *et al*., 2013; Li *et al*., 2016). Moreover, the intratumoral volume of hypoxic regions has been reported to be positively correlated with the poor clinical prognosis of patients with pancreatic cancer (Hiraoka *et al*., 2010).

Another characteristic feature of pancreatic cancer is the desmoplastic reaction. It is a remarkable fact that PCCs do not exist in isolation during disease progression. PCCs are surrounded by a dense desmoplastic stroma, which consists of pancreatic stellate cells (PSCs), immune cells, lymphatic and vascular endothelial cells, pathologically increased nerves, and extracellular matrix (ECM) (Tang *et al*., 2013). PSCs are the major cellular contributors to the desmoplastic reaction, and they are regarded as critical players in the progression of pancreatic cancer (Apte *et al*., 2004; Tang *et al*., 2013). Under normal conditions, PSCs are maintained in a relatively ‘quiescent’ state; however, PSCs can switch to an ‘activated’ phenotype, which is characterized by a tendency to synthesize certain biologically active molecules (such as α‐smooth muscle (α‐SMA), collagens, and matrix metalloproteinases (MMPs)) in response to various stimulations (Bynigeri *et al*., 2017; Masamune and Shimosegawa, 2015). Moreover, activated PSCs promote PCC proliferation, migration, and invasion and induce EMT and cancer stem cell (CSC)‐like phenotypes of PCCs via paracrine of a variety of cytokines, which results in distant metastasis, resistance to chemotherapy, and poor clinical prognosis in patients with pancreatic cancer (Hwang *et al*., 2008; Zhang *et al*., 2015). Nevertheless, the precise mechanism accounting for the tumor–stroma interactions is still unclear.

Epithelial–mesenchymal transition is a process defining the progression in which cells lose their polarized epithelial character and acquire a mesenchymal phenotype. EMT results in the loss of E‐cadherin expression and the acquisition of mesenchymal markers, such as N‐cadherin and Vimentin (Beuran *et al*., 2015). CSC‐like properties are defined as high tumorigenicity, self‐renewal, and differentiation capacities (Lee *et al*., 2008). CSCs have been identified by several putative markers, including Sox2, Oct4, Nanog, CD24, and CD133 (Luo *et al*., 2017; Zhang *et al*., 2016). Increasing evidence has indicated a close connection between EMT and CSC‐like properties (Floor *et al*., 2011; Zhou *et al*., 2017b). EMT and CSC‐like properties are regulated by a complex network of cytokines, transcription factors, and signaling pathways, and inhibition of EMT and CSC‐like properties is considered as a promising strategy for pancreatic cancer treatment (Zhou *et al*., 2017b).

Osteopontin (OPN, encoded by SPP1 gene), an integrin binding glycoprotein, has been implicated in a variety of physiological and pathophysiological processes, including bone remodeling, immune responses, and cancer progression (Castello *et al*., 2017; Ishijima *et al*., 2001). Mainly through interacting with integrin αvβ3, a member of the integrin family, OPN can activate several downstream signaling pathways, such as the PI3K/Akt, Mrk/Erk, NF‐κB, and STAT3 pathways (Chen *et al*., 2009; Urtasun *et al*., 2012). OPN is overexpressed in various types of cancer, including pancreatic cancer (Koopmann *et al*., 2004), and OPN treatment has been reported to induce malignant phenotypes in PCCs (Kolb *et al*., 2005). However, little is known about the regulation of the aberrant expression of OPN and the potential roles of OPN in the tumor–stroma interactions of pancreatic cancer.

Forkhead box protein M1 (FOXM1), a member of the Forkhead box transcription factor family, was first discovered as an oncogene, and it plays a key role in the cell‐cycle progression (Kalin *et al*., 2011; Pilarsky *et al*., 2004). Aberrant expression of FOXM1 has been observed in the majority of human solid tumors, including pancreatic cancer (Dai *et al*., 2015). Accumulating evidence has suggested that aberrant expression of FOXM1 contributes to the carcinogenesis and progression of pancreatic cancer (Cui *et al*., 2016; Quan *et al*., 2013). Interestingly, a previous study has reported that OPN induced the expression of FOXM1 in human uterine epithelial cells (Xie *et al*., 2014), indicating a potential role of OPN in regulating FOXM1 expression. Nevertheless, the relationship between OPN and FOXM1 in pancreatic cancer still remains unknown.

Our group has focused on the tumor microenvironment of pancreatic cancer in recent years (Duan *et al*., 2017; Lei *et al*., 2014; Li *et al*., 2014b). In the present study, we aimed to investigate the relationship between paracrine OPN signaling and FOXM1 and to further elucidate their contribution to the tumor–stroma interaction‐mediated EMT and stemness of tumor cells in pancreatic cancer.

## 2 Materials and methods

### 2.1 Reagents

Recombinant human OPN protein (rhOPN) and OPN blocking antibody were purchased from R&D Systems (Minneapolis, MN, USA). Integrin αvβ3 blocking antibody was obtained from Millipore (Darmstadt, Germany). Thiostrepton (TST) was purchased from MedChemExpress (Princeton, NJ, USA). MK‐2206 and U0126 were obtained from Selleckchem (Houston, TX, USA). N‐acetyl‐l‐cysteine (NAC) was purchased from Sigma (St. Louis, MO, USA). All reagents were stored according to the manufacturer's instructions.

### 2.2 Human tissue specimens, cell lines, and cell culture

Normal pancreatic tissues were obtained from patients undergoing liver transplantation at the Department of Hepatobiliary Surgery at the First Affiliated Hospital of Xi'an Jiaotong University. Ethical approval was obtained from the Ethical Committee of the First Affiliated Hospital of Xi'an Jiaotong University. All patients provided written informed consent for use of their tissue specimens. The study methodologies conformed to be standards set by the Declaration of Helsinki. PSCs were isolated from the tissues and cultured as previously described (Duan *et al*., 2017). Human pancreatic cancer cell lines (CFPAC‐1, PANC‐1, BxPC‐3, and MIA PaCa‐2) were purchased from the Type Culture Collection of the Chinese Academy of Sciences (Shanghai, China). The PANC‐1 and MIA PaCa‐2 cells were cultured in Dulbecco's modified Eagle's medium (DMEM, Gibco) with 10% fetal bovine serum (FBS) and antibiotics (1% penicillin and streptomycin), CFPAC‐1 cells were cultured in Iscove's Modified Dulbecco's Medium (IMDM, Gibco) with 10% FBS and antibiotics, and BxPC‐3 cells were cultured in RPMI‐1640 (Gibco) medium with 10% FBS and antibiotics. Cells were cultured in a humidified atmosphere containing 5% CO_2_ at 37 °C. In experiments designed to assess the roles of hypoxia, cells were first cultured in normoxic conditions to obtain the desired subconfluence level (65–70%) and then were incubated in strictly controlled hypoxic conditions (1% O_2_).

### 2.3 Conditioned medium of PSCs and enzyme‐linked immunosorbent assay

Pancreatic stellate cells (5 × 10^6^) were grown in 75‐mL cell culture flasks until reaching 80–90%confluence and were then incubated with serum‐free DMEM/F‐12 for 72 h. Collected culture medium was centrifuged at about 380 *g* for 5 min and filtered before being stored at −80 °C for further use. The culture medium was mixed with serum‐free medium in the following experiments to assess the potential effects of PSC secretions on pancreatic cancer cells. The production of OPN in the supernatants was quantified using a human OPN ELISA kit (Boster, Wuhan, China) according to the manufacturer's recommendations.

### 2.4 Immunofluorescence staining

Cells were fixed in 4% paraformaldehyde for 15 min, washed with PBS containing 0.1% Tween‐20, permeabilized using 0.3% Triton X‐100, blocked with 5% BSA in PBS, and then incubated first with the primary antibodies at 4 °C overnight and subsequently with fluorescently labeled secondary antibodies from Jackson ImmunoResearch Laboratories (West Grove, PA, USA) at room temperature for 1 h. Nuclei were stained for 5 min using DAPI. Slides were mounted and examined using a Zeiss Instruments confocal microscope.

### 2.5 Measurement of intracellular ROS levels

Intracellular ROS levels were measured by the oxidation‐sensitive fluorescent dye DCFH‐DA. Five minutes before the end of the incubation, the cells were incubated with 10 μM DCFH‐DA (Invitrogen, Carlsbad, CA, USA) for approximately 30 min. After washing 3 times with PBS, the cells were lysed in 1 mL of RIPA buffer and analyzed immediately by fluorimetric analysis at 510 nm. The final results were normalized to the total protein content.

### 2.6 Quantitative real‐time RT‐PCR

Total RNA was extracted using a Fastgen1000 RNA isolation system (Fastgen, Shanghai, China) following the instructions in the manufacturer's protocol. Then, complementary DNA (cDNA) was synthesized from RNA using a Prime Script RT reagent kit (TaKaRa, Dalian, China). Quantitative real‐time RT‐PCR was utilized to examine the mRNA expression of target genes. The primer sequences for the target genes and β‐actin are listed in Table S1. The expression of each target gene was determined with β‐actin as the normalization control. The relative gene expression was calculated using the 2^−∆∆Ct^ method.

### 2.7 Western blot analysis

Total proteins were extracted by RIPA lysis buffer (Beyotime, Guangzhou, China), and the concentration of proteins was determined through a BCA protein assay kit (Pierce, Rockford, IL, USA) following the instructions in the manufacturer's protocol. The proteins were then subjected to SDS/PAGE using a 10% polyacrylamide gel with a 5% stacking gel and then transferred to polyvinylidene difluoride (PVDF) membranes. The membranes were blocked with 5% fat‐free milk in Tris‐buffered saline‐Tween (TBS‐T) for 2 h and then incubated with the primary antibodies overnight at 4 °C. Following the incubation with the secondary horseradish peroxidase (HRP)‐conjugated antibodies for 2 h at room temperature, the immunocomplexes were detected using the enhanced chemiluminescence kit and the Molecular Imager ChemiDoc XRS System (Bio‐Rad, Hercules, CA, USA). The antibodies utilized in our studies are listed in Table S2.

### 2.8 Wound healing assay

The migratory ability of cancer cells was evaluated by the wound healing assay. The cells were seeded in fibronectin‐coated 6‐well plates. After the cells reached the appropriate confluence, a sterile pipette tip was used to produce a wound line between the cells. The cells were washed 3 times with PBS and cultured in FBS‐free medium. Images of the same locations were taken at 0 h and 24 h postwounding under a Nikon Diaphot TMD inverted microscope. The borders were marked by red lines, and the relative distance traveled by the leading edge from 0 to 24 h was determined for the final results.

### 2.9 Matrigel invasion assay

The invasive capacity of cancer cells was examined by the Matrigel invasion assay. The upper surface of the membrane was coated with Matrigel (BD Biosciences, Franklin Lakes, NJ, USA). The cells (5 × 10^4^) were suspended in FBS‐free medium and then seeded in the upper chamber. In the lower chamber, DMEM supplemented with 10% FBS was added to generate a gradient to drive cell migration. Forty‐eight hours later, the medium was aspirated, and noninvasive cells in the upper chamber were removed by a cotton swab. The invading cells were then fixed in 4% paraformaldehyde and stained by crystal violet. The number of cells on the membrane was determined by counting under a microscope.

### 2.10 Tumorsphere formation assay

A tumorsphere formation assay was performed to evaluate the sphere‐forming ability of the cancer cells. The cells were plated into 6‐well ultralow attachment plates (Corning, Corning, NY, USA) at a density of 5000 cells/well in serum‐free DMEM/F12 medium (Gibco); subsequently, 10 ng·mL^−1^ human EGF, 10 ng·mL^−1^ human FGF, and 2% B27 (Invitrogen, Carlsbad, CA, USA) were added. Then, the cells were incubated at 37 °C and 5% CO_2_. After 2 weeks, the plates were analyzed for tumorsphere formation, and the number of tumorspheres was counted under a microscope.

### 2.11 Human tissue specimens and histological analysis

Seventy‐five pancreatic cancer tissues and twenty‐five normal pancreatic tissues with corresponding clinical information were collected from patients who were diagnosed with pancreatic cancer at the Department of Hepatobiliary Surgery, the First Affiliated Hospital of Xi'an Jiaotong University. The patients did not receive chemotherapy or radiation therapy before surgery. Ethical approval was obtained from the Ethical Committee of the First Affiliated Hospital of Xi'an Jiaotong University. All patients provided written informed consent for use of their tissue specimens. The study methodologies conformed to be standards set by the Declaration of Helsinki. H&E staining and immunohistochemical (IHC) staining for OPN and FOXM1 were performed using standard procedures as previously described (Duan *et al*., 2017). All the slides were evaluated and scored by two independent pathologists who were blinded to the clinicopathological data and reached a consensus. The percentages of positive tumor or stromal cells were categorized as follows: 0 = <10%, 1 = 10–25%, 2 = 25–50%, 3 = 50–75%, and 4 =  >75%. The staining intensity was scored as follows: 0 = no staining, 1 = light brown, 2 = brown, and 3 = dark brown. The IHC score for the percentage of positive tumor or stromal cells and staining intensity were multiplied to achieve a weighted score for each case, and cases with scores ≥3 were defined as positive.

### 2.12 Analysis of the expression level and prognostic value of candidate genes in pancreatic cancer with the Cancer Genome Atlas data

Analysis of the expression level and prognostic value of candidate genes in pancreatic cancer was based on Gene Expression Profiling Interactive Analysis (GEPIA) (Tang *et al*., 2017), which can analyze the RNA sequencing expression data of various types of cancer for comparison between tumor tissues and normal tissues from the Cancer Genome Atlas (TCGA) database according to the standard processing pipeline.

### 2.13 Statistical analysis

The data shown in this study are presented as the mean ± SD. Comparisons between the groups were conducted using Student's *t*‐test or one‐way ANOVA analysis of variance with Tukey's multiple comparisons test. The correlations between expression of OPN or FOXM1 and clinical characteristics were analyzed by Pearson's chi‐square tests with Yates continuity correction. All statistical analysis was performed by SPSS 20.0 (SPSS Inc., Chicago, IL, USA). Statistical significance was defined as a two‐sided *P*‐value < 0.05 unless specifically indicated.

## 3 Results

### 3.1 Hypoxia‐driven PSC activation is ROS‐dependent

**Figure 1:**
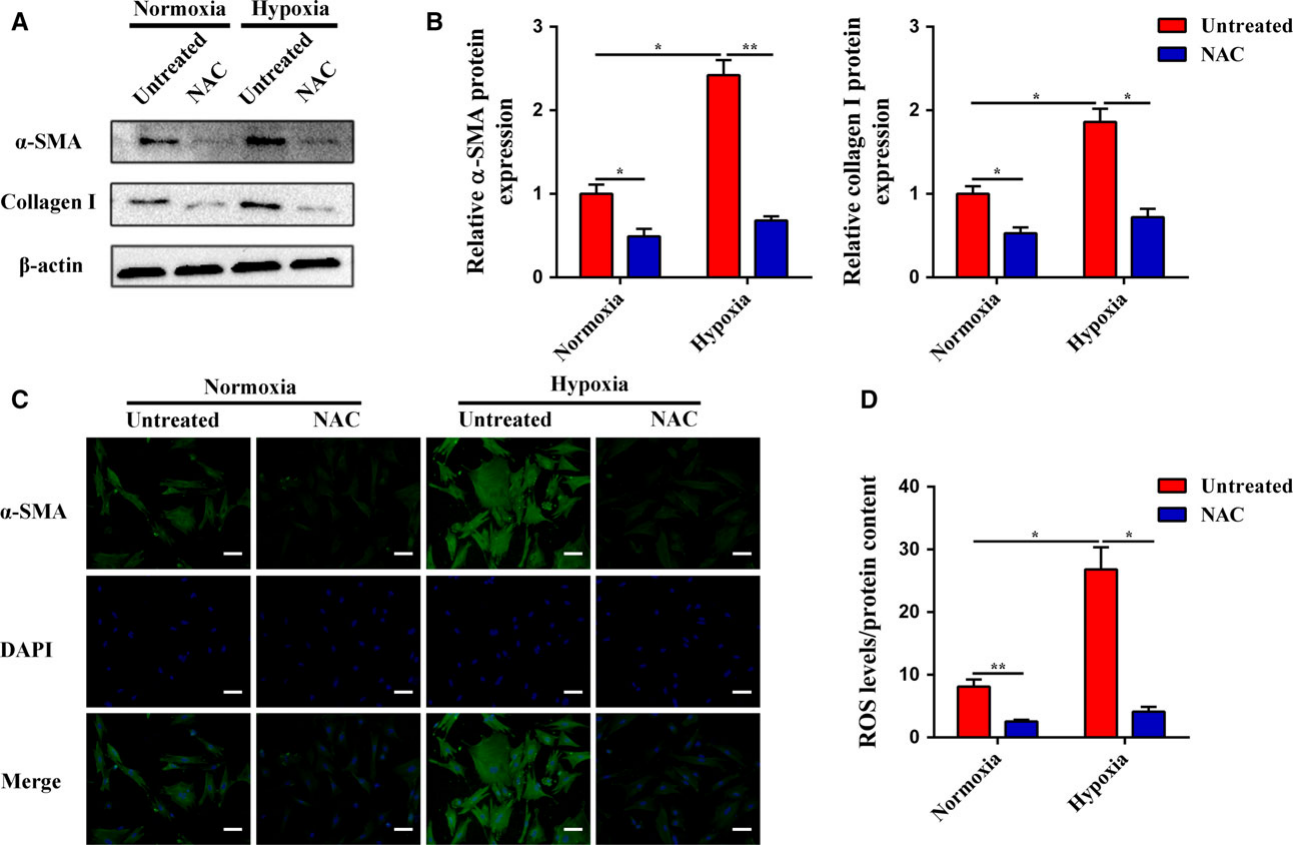
Hypoxia‐driven PSC activation is ROS‐dependent. (A and B) Subconfluent PSCs were cultured in normoxia or hypoxia with or without NAC, and then, the protein expression levels of α‐SMA and collagen I were determined by western blot analysis. β‐Actin was used as an internal control. (C) PSCs were treated as in (A), and the α‐SMA expression was analyzed by immunofluorescence staining. The green signal represents α‐SMA staining, and nuclear DNA staining by DAPI is shown in blue. The scale bar represents 50 μm. (D) PSCs were treated as in (A), and the ROS production was evaluated by DCFH‐DA and normalized based on the total protein content. All data are shown as the mean ± SD of at least three independent experiments. One‐way ANOVA and Tukey's multiple comparisons test were performed to determine significance. *P < 0.05, **P < 0.01.

Hypoxia is believed to play an important role in PSC activation, contributing to tumor invasiveness and metastatic spreading (Bynigeri *et al*., 2017). In the present study, we observed that hypoxia promoted PSC activation, as revealed by increased α‐SMA and type I collagen expression (Fig. 1A,B). Immunofluorescence staining also showed that PSCs were activated under hypoxia with increased α‐SMA expression (Fig. 1C). Hypoxia has already been correlated with a state of oxidative stress, and studies have demonstrated that ROS induction is one of the most common regulation mechanisms under hypoxic conditions. Hence, we assayed the ROS produced by PSCs in response to hypoxia. As shown in Fig. 1D, the ROS levels were significantly increased under hypoxia compared to those under normoxia. However, NAC, a ROS scavenger, abolished the ROS generation and attenuated the PSC activation induced by hypoxia, which was consistent with our previous results (Lei *et al*., 2014). Altogether, these data indicate that ROS play a key role in hypoxia‐driven PSC activation.

### 3.2 Hypoxia‐driven ROS‐induced PSC activation increases the expression and secretion of OPN

**Figure 2:**
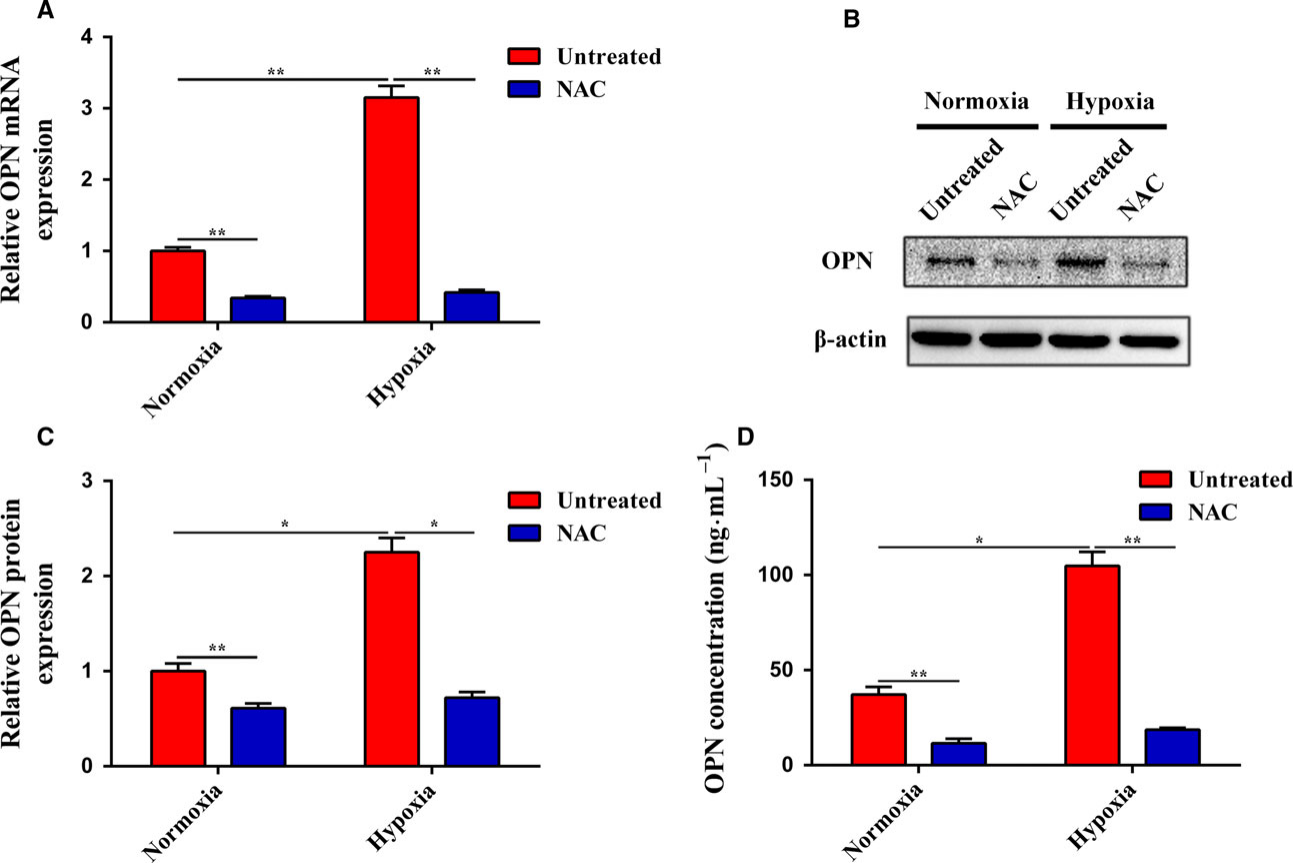
Hypoxia‐driven ROS‐induced PSC activation increases the expression and secretion of OPN. (A) Subconfluent PSCs were cultured in normoxia or hypoxia with or without NAC, and then, the mRNA expression of OPN was analyzed by quantitative real‐time RT‐PCR. (B and C) PSCs were treated as in (A), and the protein expression of OPN was determined by western blot analysis. β‐Actin was used as an internal control. (D) PSCs were treated as in (A), and the OPN secretion was evaluated by ELISA. All data are shown as the mean ± SD of at least three independent experiments. One‐way ANOVA and Tukey's multiple comparisons test were performed to determine significance. *P < 0.05, **P < 0.01.

We first assayed the expression of OPN in response to hypoxia. As shown in Fig. 2A–C, OPN was upregulated in activated PSCs driven by hypoxia compared to PSCs under normoxia at both the mRNA and protein levels. Next, we performed enzyme‐linked immunosorbent assay to determine whether the high‐level expression of OPN in activated PSCs driven by hypoxia was also increased in the cell supernatants. We found that the concentration of OPN was significantly higher in the supernatants of activated PSCs driven by hypoxia than in those of PSCs under normoxia (Fig. 2D). However, NAC treatment abrogated the hypoxia‐induced overexpression and secretion of OPN, which demonstrated the critical roles of ROS in the regulation of OPN under hypoxia.

### 3.3 Expression of OPN and integrin αvβ3 in PSCs and PCCs

**Figure 3:**
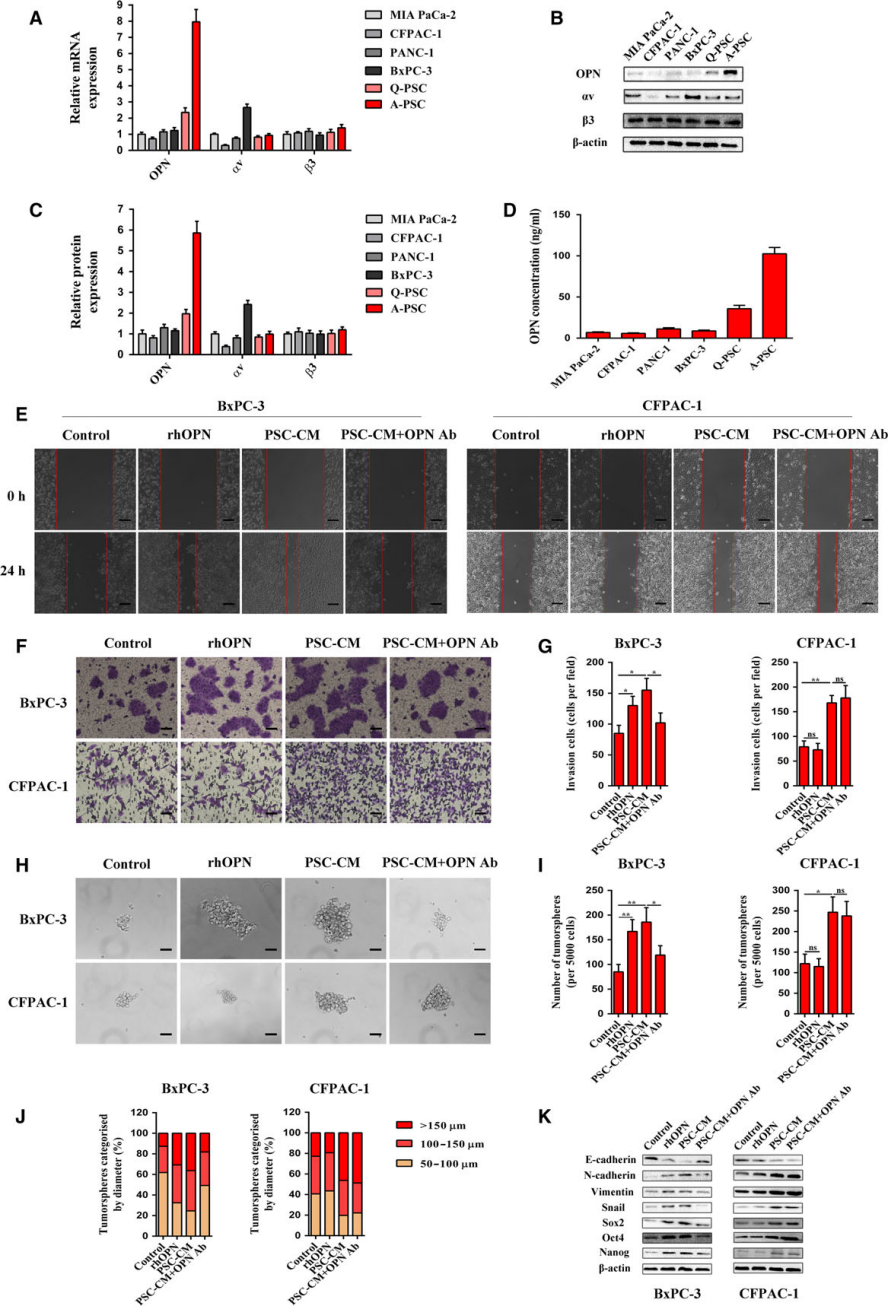
Hypoxia‐driven paracrine OPN signaling promotes EMT and CSC‐like properties in PCCs. (A) The mRNA expression levels of OPN, the αv subunit of integrin, and the β3 subunit of integrin in 4 pancreatic cancer cell lines and PSCs were analyzed by quantitative real‐time RT‐PCR. (B and C) The protein expression levels of OPN, the αv subunit of integrin, and the β3 subunit of integrin in 4 pancreatic cancer cell lines and PSCs were determined by western blot analysis. β‐Actin was used as an internal control. (D) The cell supernatants of 4 pancreatic cancer cell lines and PSCs were collected, and the OPN concentration was measured by ELISA. (E) Representative images from the wound healing assay after rhOPN, PSC‐CM, or PSC‐CM + OPN Ab treatments in BxPC‐3 and CFPAC‐1 cells. Images were obtained at 0 h and 24 h. The scale bar represents 100 μm. (F and G) Representative images of the Matrigel invasion assay after rhOPN, PSC‐CM, or PSC‐CM + OPN Ab treatments in BxPC‐3 and CFPAC‐1 cells. The invasive cells were quantified by counting the number of cells in 10 random fields. The scale bar represents 50 μm. (H, I and J) Representative images of the tumorsphere formation assay after rhOPN, PSC‐CM, or PSC‐CM + OPN Ab treatments in BxPC‐3 and CFPAC‐1 cells. The number of tumorspheres was counted and plotted, and the percentage of tumorspheres with diameters of 50–100 μm, 100–150 μm, or >150 μm was calculated and plotted. The scale bar represents 50 μm. (K) The protein expression levels of EMT and CSC markers after rhOPN, PSC‐CM, or PSC‐CM + OPN Ab treatments were determined by western blot analysis in BxPC‐3 and CFPAC‐1 cells. β‐Actin was used as an internal control. Q‐PSC: Quiescent pancreatic stellate cell. A‐PSC: Activated pancreatic stellate cell. All data are shown as the mean ± SD of at least three independent experiments. One‐way ANOVA and Tukey's multiple comparisons test were performed to determine significance. *P < 0.05, **P < 0.01, ns: no significance.

Integrin αvβ3 has been reported to be a potential receptor of OPN (Chen *et al*., 2009; Urtasun *et al*., 2012). Thus, we first analyzed the expression levels of OPN and integrin αvβ3 in 4 pancreatic cancer cell lines and PSCs. As shown in Fig. 3A, OPN was mainly expressed in PSCs, especially in activated PSCs driven by hypoxia, while the β3 subunit of integrin was expressed in both tumor cells and PSCs. However, the αv subunit of integrin was differentially expressed in the above tested cells. Specifically, BxPC‐3 cells showed the highest expression, whereas CFPAC‐1 cells had little expression of the αv subunit of integrin. The other cells showed moderate expression of the αv subunit of integrin. The similar expression patterns were confirmed by western blot analysis (Fig. 3B,C). Moreover, the concentration of OPN was significantly higher in the supernatants of activated PSCs driven by hypoxia than in those of the other tumor cells or PSCs under normoxia, indicating that the primary source of OPN in pancreatic cancer is activated PSCs (Fig. 3D). Together, these data suggest a possible crosstalk of paracrine OPN/integrin αvβ3 signaling between PSCs and PCCs.

### 3.4 Hypoxia‐driven paracrine OPN signaling promotes the EMT and CSC‐like properties in PCCs

To evaluate whether activated PSCs driven by hypoxia had any impact on the malignant phenotypes of PCCs, BxPC‐3 and CFPAC‐1 cells were chosen and subjected to the following experiments. Both recombinant human OPN (rhOPN) and PSC culture medium supernatant (PSC‐CM) treatments for 24 h increased the migratory and invasive capacities of BxPC‐3 cells, as confirmed by the wound healing assay and the Matrigel invasion assay (Fig. 3E–G). Moreover, the assay of tumorsphere formation, which is regarded as a representative trait of CSCs (Abel and Simeone, 2013), was utilized to evaluate the sphere‐forming ability of PCCs. The results showed that both the number and the size of tumorspheres in BxPC‐3 cells were surprisingly increased after rhOPN or PSC‐CM treatment (Fig. 3H–J). However, the effects of PSC‐CM could be partly reversed by OPN‐neutralizing antibody, demonstrating the important roles of OPN in hypoxia‐driven tumor–stroma interactions. In addition, rhOPN or PSC‐CM treatment induced the expression of EMT and CSC markers in BxPC‐3 cells, as revealed by decreased E‐cadherin expression and increased N‐cadherin, vimentin, Snail, Sox2, Oct4, and Nanog expression (Fig. 3K). Similarly, these effects of PSC‐CM were also partly hindered by OPN‐neutralizing antibody. By contrast, rhOPN failed to induce EMT and CSC‐like properties in CFPAC‐1 cells similar to those induced by PSC‐CM, and OPN‐neutralizing antibody was unable to attenuate the effects of PSC‐CM, indicating that integrin αvβ3 may participate in paracrine OPN signaling based on its different expression patterns of these two cell lines.

### 3.5 Integrin αvβ3 mediates the effects of paracrine OPN signaling

**Figure 4:**
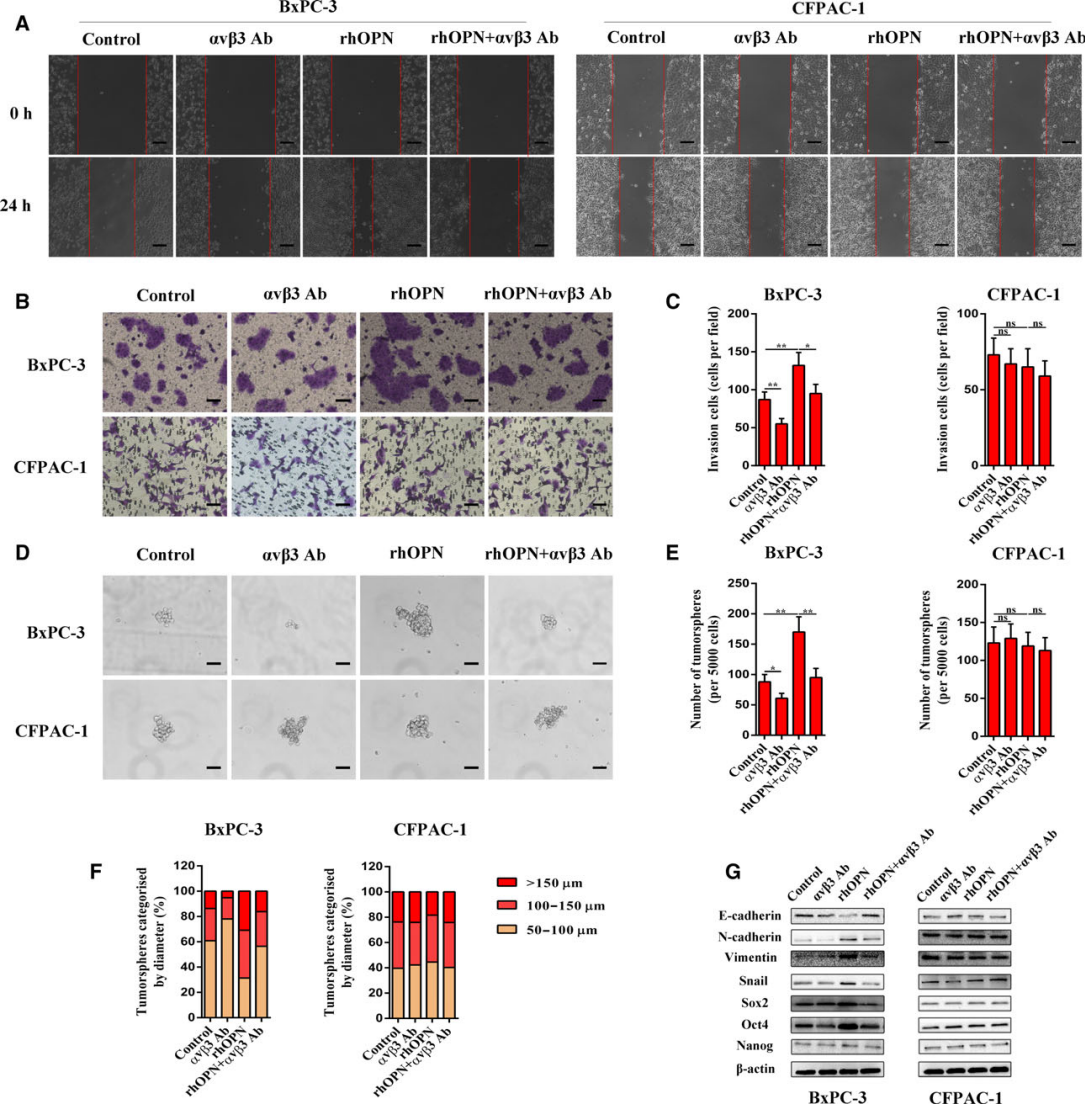
Integrin αvβ3 mediates the effects of paracrine OPN signaling on EMT and CSC‐like properties in PCCs. (A) Representative images from the wound healing assay after αvβ3 Ab, rhOPN, or rhOPN + αvβ3 Ab treatments in BxPC‐3 and CFPAC‐1 cells. Images were obtained at 0 h and 24 h. The scale bar represents 100 μm. (B and C) Representative images of the Matrigel invasion assay after αvβ3 Ab, rhOPN, or rhOPN + αvβ3 Ab treatments in BxPC‐3 and CFPAC‐1 cells. The invasive cells were quantified by counting the number of cells in 10 random fields. The scale bar represents 50 μm. (D, E, and F) Representative images from the tumorsphere formation assay after αvβ3 Ab, rhOPN, or rhOPN + αvβ3 Ab treatments in BxPC‐3 and CFPAC‐1 cells. The number of tumorspheres was counted and plotted, and the percentage of tumorspheres with diameters of 50–100 μm, 100–150 μm, or >150 μm was calculated and plotted. The scale bar represents 50 μm. (G) The protein expression levels of EMT and CSC markers after αvβ3 Ab, rhOPN, or rhOPN + αvβ3 Ab treatments were determined by western blot analysis in BxPC‐3 and CFPAC‐1 cells. β‐Actin was used as an internal control. All data are shown as the mean ± SD of at least three independent experiments. One‐way ANOVA and Tukey's multiple comparisons test were performed to determine significance. *P < 0.05, **P < 0.01, ns: no significance.

To further elucidate the molecular mechanisms involved in paracrine OPN signaling, a specific integrin αvβ3‐neutralizing antibody was used in the following experiments. In BxPC‐3 cells, integrin αvβ3‐neutralizing antibody was able to impede the rhOPN‐induced migratory and invasive capacities (Fig. 4A–C), as well as the sphere‐forming abilities (Fig. 4D–F). Similarly, integrin αvβ3‐neutralizing antibody also attenuated the rhOPN‐induced EMT and CSC markers, as confirmed by western blotting (Fig. 4G). However, neither rhOPN nor integrin αvβ3‐neutralizing antibody exerted effects on the EMT and CSC‐like properties of CFPAC‐1 cells. These findings suggest that the effects of paracrine OPN signaling on PCCs are predominantly mediated by integrin αvβ3.

### 3.6 FOXM1 mediates the effects of the OPN/integrin αvβ3 axis

**Figure 5:**
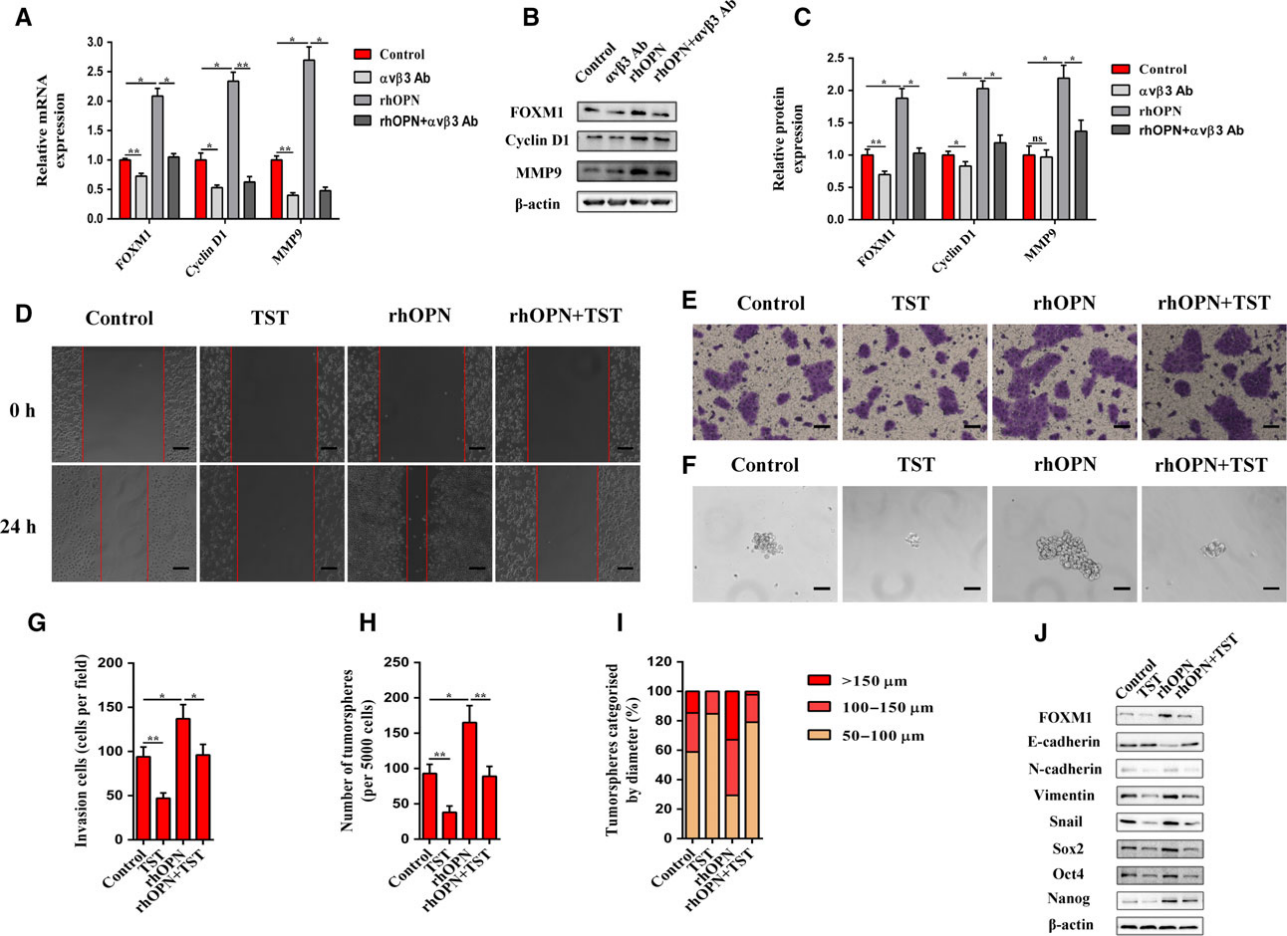
FOXM1 mediates the effects of the OPN/integrin αvβ3 axis on EMT and CSC‐like properties in PCCs. (A) The mRNA expression levels of FOXM1 and its downstream targets after αvβ3 Ab, rhOPN, or rhOPN + αvβ3 Ab treatments were determined by quantitative real‐time RT‐PCR in BxPC‐3 cells. (B and C) The protein expression levels of FOXM1 and its downstream targets after αvβ3 Ab, rhOPN, or rhOPN + αvβ3 Ab treatments were determined by western blot analysis in BxPC‐3 cells. β‐Actin was used as an internal control. (D) Representative images from the wound healing assay after TST, rhOPN, or rhOPN + TST treatments in BxPC‐3 cells. Images were obtained at 0 h and 24 h. The scale bar represents 100 μm. (E and G) Representative images from the Matrigel invasion assay after TST, rhOPN, or rhOPN + TST treatments in BxPC‐3 cells. The invasive cells were quantified by counting the number of cells in 10 random fields. The scale bar represents 50 μm. (F, H, and I) Representative images from the tumorsphere formation assay after TST, rhOPN, or rhOPN + TST treatments in BxPC‐3 cells. The number of tumorspheres was counted and plotted, and percentage of tumorspheres with diameters of 50–100 μm, 100–150 μm, or >150 μm was calculated and plotted. The scale bar represents 50 μm. (J) The protein expression levels of EMT and CSC markers after TST, rhOPN, or rhOPN + TST treatments were determined by western blot analysis in BxPC‐3 cells. β‐Actin was used as an internal control. All data are shown as the mean ± SD of at least three independent experiments. One‐way ANOVA and Tukey's multiple comparisons test were performed to determine significance. *P < 0.05, **P < 0.01, ns: no significance.

To further determine the downstream effector of the paracrine OPN/integrin αvβ3 axis and to explore the potential roles of FOXM1 in OPN‐induced malignant phenotypes in PCCs, we first evaluated FOXM1 and its downstream targets (cyclin D1 and MMP‐9) (Wang *et al*., 2007) after rhOPN treatment or integrin αvβ3 blockage. The results showed that FOXM1 expression was elevated after rhOPN stimulation, which was hampered by integrin αvβ3‐neutralizing antibody treatment at both the mRNA and protein levels in BxPC‐3 cells. Moreover, rhOPN and integrin αvβ3‐neutralizing antibody exerted similar influences on downstream targets of FOXM1, whose increased levels under rhOPN stimulation were dampened upon integrin αvβ3‐neutralizing antibody treatment (Fig. 5A–C). With the aim of further evaluating whether FOXM1 is the downstream effector of the paracrine OPN/integrin αvβ3 axis, BxPC‐3 cells were pretreated with TST, a reagent that targets FOXM1 (Weiler *et al*., 2017), for 24 h and then incubated with rhOPN for another 24 h. As expected, rhOPN treatment increased the migratory and invasive capacities, as well as sphere‐forming abilities of BxPC‐3 cells (Fig. 5D–I). The effects of rhOPN treatment were dramatically abolished by TST, which illustrated the critical roles of FOXM1 in the paracrine OPN/integrin αvβ3 axis. Analogously, the rhOPN‐induced EMT and CSC markers of BxPC‐3 cells were decreased by TST (Fig. 5J).

### 3.7 OPN/integrin αvβ3 axis activates Akt and Erk to increase the expression of FOXM1 in PCCs

**Figure 6:**
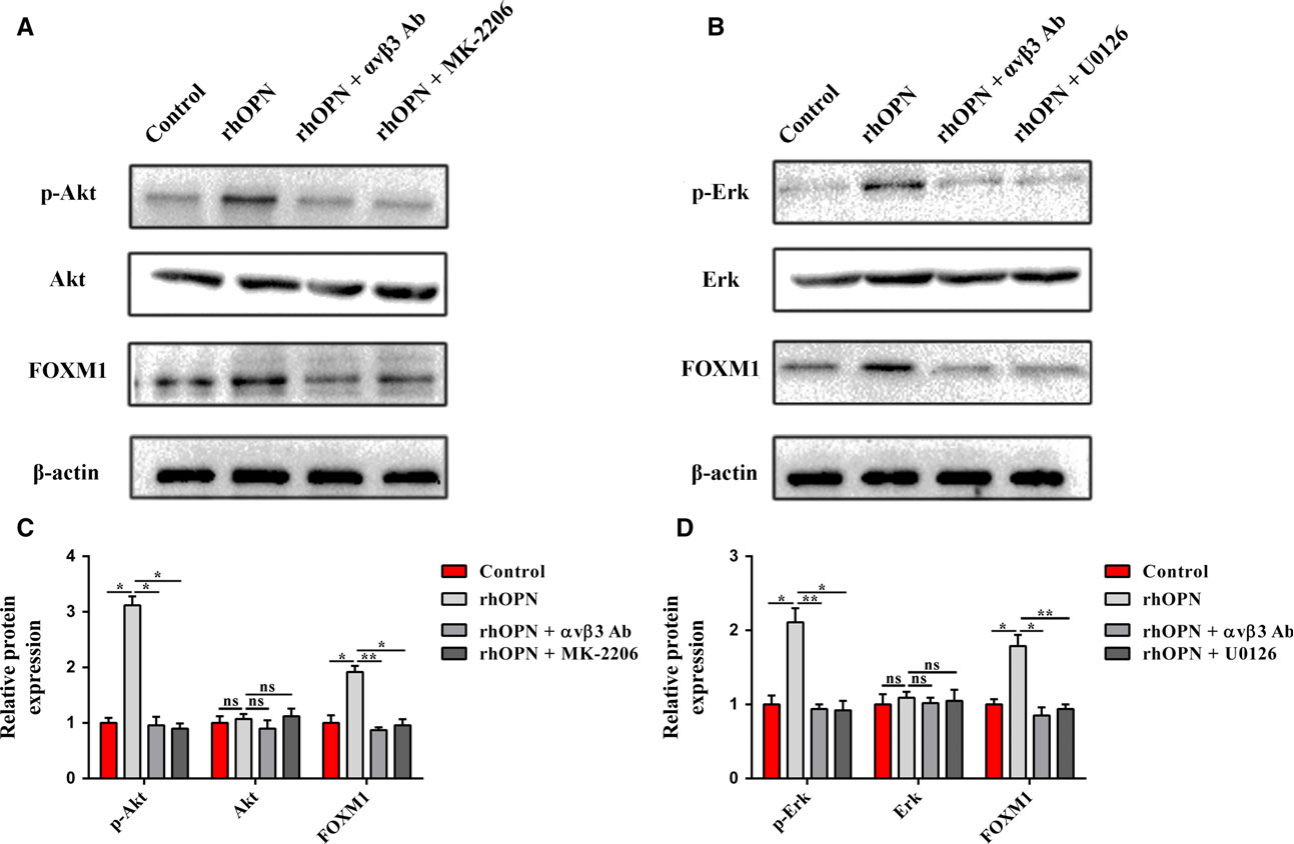
The OPN/integrin αvβ3 axis induces FOXM1 expression by activating the Akt and Erk signaling pathways in PCCs. (A and C) Western blot analysis showed that rhOPN increased Akt phosphorylation to induce FOXM1 expression in BxPC‐3 cells, while this effect was antagonized by blocking integrin αvβ3 or inhibiting Akt. β‐Actin was used as an internal control. (B and D) Western blot analysis showed that rhOPN increased Erk phosphorylation to induce FOXM1 expression in BxPC‐3 cells, while this effect was antagonized by blocking integrin αvβ3 or inhibiting Erk. β‐Actin was used as an internal control. All data are shown as the mean ± SD of at least three independent experiments. One‐way ANOVA and Tukey's multiple comparisons test were performed to determine significance. *P < 0.05, **P < 0.01, ns: no significance.

To further clarify the mechanisms of the paracrine OPN/integrin αvβ3 axis‐induced FOXM1 expression, we assessed the expression and phosphorylation of Akt and Erk in BxPC‐3 cells by western blotting. As shown in Fig. 6A–D, rhOPN treatment increased both Akt and Erk phosphorylation, but these effects were obviously suppressed by integrin αvβ3‐neutralizing antibody. In addition, the enhanced Akt and Erk phosphorylation upon rhOPN treatment was nearly abolished by pretreating BxPC‐3 cells with MK‐2206 (a selective Akt inhibitor) or U0126 (a selective Erk inhibitor). To confirm the involvement of Akt and Erk in the paracrine OPN/integrin αvβ3 axis‐induced FOXM1 expression, we further measured the FOXM1 expression in response to rhOPN after pretreating BxPC‐3 cells with Akt or Erk inhibitor. Unsurprisingly, the results demonstrated that the Akt or Erk inhibitor each attenuated the paracrine OPN/integrin αvβ3 axis‐induced FOXM1 expression in BxPC‐3 cells.

### 3.8 Expression and prognostic value of OPN and FOXM1 in pancreatic cancer

**Figure 7:**
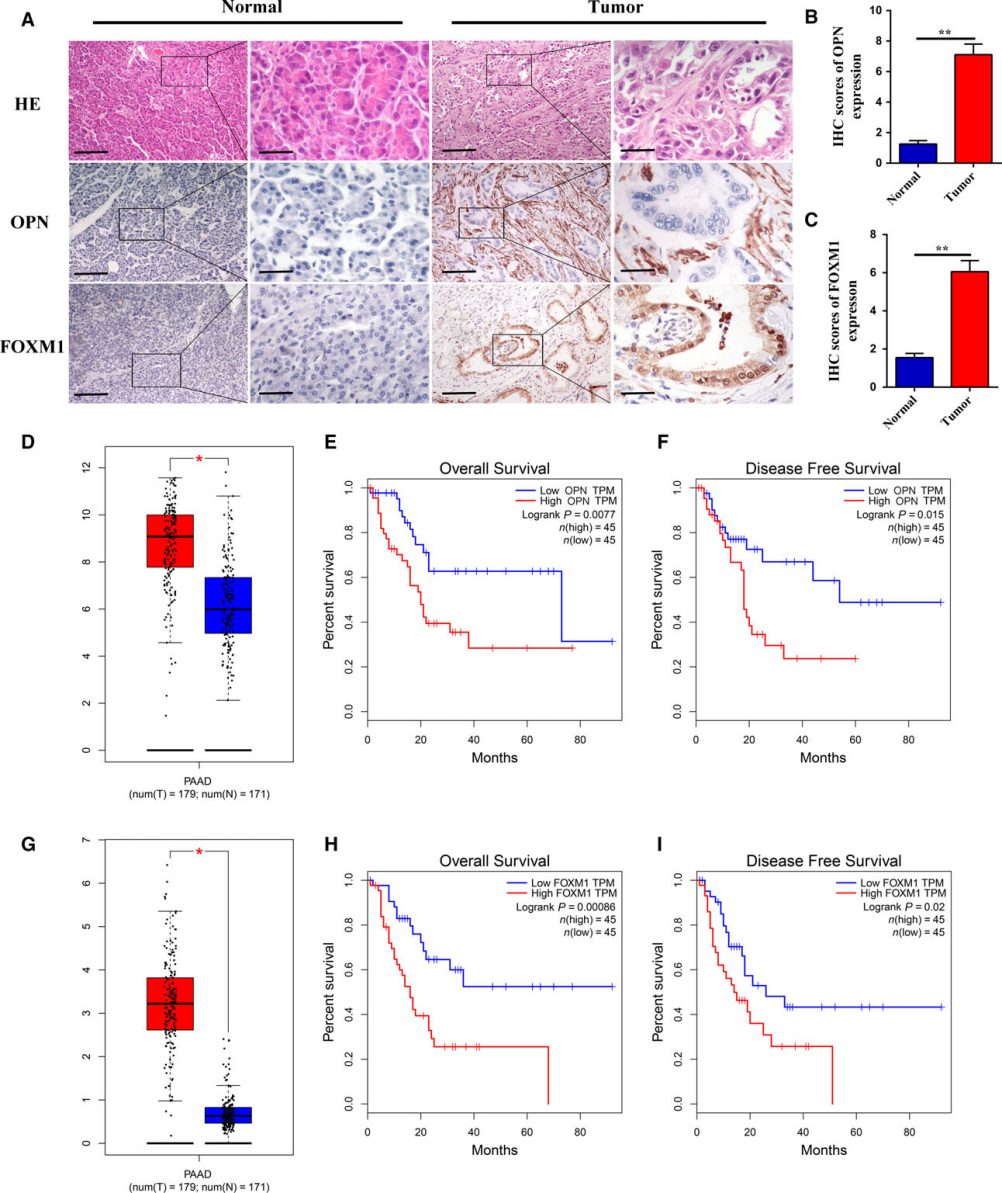
Expression and prognostic value of OPN and FOXM1 in pancreatic cancer. (A) Representative images showing the absence of OPN or FOXM1 expression in normal pancreatic tissues and the presence of OPN expression in stromal cells or FOXM1 expression in tumor cells of pancreatic cancer tissues. The scale bar represents 400 μm for low magnification field and 100 μm for high magnification field. (B and C) The IHC scores of OPN (B) and FOXM1 (C) expression in normal pancreatic tissues (*n* = 25) and pancreatic cancer tissues (*n* = 75). (D and G) The expression of OPN (D) and FOXM1 (G) was upregulated in pancreatic cancer tissues compared to that in normal tissues. The red bar shows the tumor tissues, and the blue bar indicates the normal tissues. (E and H) The expression of OPN (E) and FOXM1 (H) was negatively correlated with pancreatic cancer patients’ OS. The red curve represents patients with a high level of OPN or FOXM1 expression (Top 25%), while the blue curve represents patients with a low level of OPN or FOXM1 expression (Bottom 25%). (F and I) The expression of OPN (F) and FOXM1 (I) was negatively correlated with pancreatic cancer patients’ DFS. The red curve represents patients with a high level of OPN or FOXM1 expression (top 25%), while the blue curve represents patients with a low level of OPN or FOXM1 expression (bottom 25%). These figures were derived from GEPIA. PAAD: Pancreatic adenocarcinoma. TPM: Transcripts per kilobase million. All data are shown as the mean ± SD of at least three independent experiments. Student's t‐test was performed to determine significance. *P < 0.05, **P < 0.01.

Based on our preliminary *in vitro* data, we further explored the clinical value of OPN and FOXM1 in pancreatic cancer patients. First, the expression levels of OPN and FOXM1 were assessed in 75 pancreatic cancer tissues and 25 normal pancreatic tissues by IHC. As shown in Fig. 7A–C, strong OPN expression in stromal cells adjacent to tumor cells was observed in pancreatic cancer tissues, whereas rare staining events were noted in the stroma and pancreatic ductal cells of normal pancreatic tissues. As for FOXM1, intense staining was detected in the cytoplasm and nucleus of tumor cells in pancreatic cancer tissues, and cells exhibited a predominantly nuclear localization pattern. However, FOXM1 expression was weak or not detected in normal pancreatic tissues. We then investigated the relationship between the expression of these two biomarkers in tumor cells or stromal cells and clinical characteristics. The results showed that OPN expression in stromal cells was significantly correlated with the tumor‐node‐metastasis (TNM) stage (*P* = 0.011) and pM status (*P* = 0.045, Table 1). Moreover, FOXM1 expression in tumor cells was found to be obviously associated with pT status (*P* = 0.014) and marginally significant with pM status (*P* = 0.095, Table 2). However, no correlation was established between OPN expression in tumor cells or FOXM1 expression in stromal cells and clinical characteristics. Next, we decided to validate our findings using TCGA data. According to the results derived from GEPIA, we observed that the expression levels of OPN and FOXM1 were significantly upregulated in pancreatic cancer tissues compared to normal pancreatic tissues (Fig. 7D and G). In addition, the expression levels of these two genes were found to be remarkably correlated with the OS and DFS of patients with pancreatic cancer (Fig. 7E,F,H, and I). Specifically, high expression of these two genes might be a risk factor affecting patient survival, while low expression corresponded to a longer survival rate and time. These results indicated that overexpression of OPN and FOXM1 might play contributory roles in the carcinogenesis and progression of pancreatic cancer, suggesting that OPN and FOXM1 could be considered as alternative diagnostic and prognostic biomarkers in pancreatic cancer.

**Table 1:** Correlation between expression of OPN in tumor/stromal cells and clinical characteristics in pancreatic cancer

Variables	Cases	OPN expression in tumor cells		*P*‐value	OPN expression in stromal cells		*P*‐value
		Negative (%)	Positive (%)		Negative (%)	Positive (%)	
Gender							
Male	43	34 (79.1)	9 (20.9)	0.815	13 (30.2)	30 (69.8)	0.618
Female	32	26 (81.3)	6 (18.7)		8 (25.0)	24 (75.0)	
Age							
≤60	24	17 (70.8)	7 (29.2)	0.293	7 (29.2)	17 (70.8)	0.877
>60	51	43 (84.3)	8 (15.7)		14 (27.5)	37 (72.5)	
TNM stage (AJCC)							
I + II	56	46 (82.1)	10 (17.9)	0.642	20 (35.7)	36 (64.3)	0.011
III + IV	19	14 (73.7)	5 (26.3)		1 (5.3)	18 (94.7)	
pT status							
T1 + T2	15	13 (86.7)	2 (13.3)	0.718	7 (46.7)	8 (53.3)	0.139
T3 + T4	60	47 (78.3)	13 (21.7)		14 (23.3)	46 (76.7)	
pN status							
N0	52	44 (84.6)	8 (15.4)	0.234	16 (30.8)	36 (69.2)	0.422
N1	23	16 (69.6)	7 (30.4)		5 (21.7)	18 (78.3)	
pM status							
M0	63	52 (82.5)	11 (17.5)	0.386	21 (33.3)	42 (66.7)	0.045
M1	12	8 (66.7)	4 (33.3)		0 (0.0)	12 (100.0)	

**Table 2:** Correlation between expression of FOXM1 in tumor/stromal cells and clinical characteristics in pancreatic cancer

Variables	Cases	FOXM1 expression in tumor cells		*P*‐value	FOXM1 expression in stromal cells		*P*‐value
		Negative (%)	Positive (%)		Negative (%)	Positive (%)	
Gender							
Male	43	12 (27.9)	31 (72.1)	0.248	40 (93.0)	3 (7.0)	0.411
Female	32	13 (40.6)	19 (59.4)		27 (84.4)	5 (15.6)	
Age							
≤60	24	6 (25.0)	18 (75.0)	0.294	22 (91.7)	2 (8.3)	0.962
>60	51	19 (37.3)	32 (62.7)		45 (88.2)	6 (11.8)	
TNM stage (AJCC)							
I + II	56	21 (37.5)	35 (62.5)	0.189	51 (91.1)	5 (8.9)	0.684
III + IV	19	4 (21.1)	15 (78.9)		16 (84.2)	3 (15.8)	
pT status							
T1 + T2	15	9 (60.0)	6 (40.0)	0.014	14 (93.3)	1 (6.7)	0.925
T3 + T4	60	16 (26.7)	44 (73.3)		53 (88.3)	7 (11.7)	
pN status							
N0	52	19 (36.5)	33 (63.5)	0.376	48 (92.3)	4 (7.7)	0.396
N1	23	6 (26.1)	17 (73.9)		19 (82.6)	4 (17.4)	
pM status							
M0	63	24 (38.1)	39 (61.9)	0.095	57 (90.5)	6 (9.5)	0.822
M1	12	1 (8.3)	11 (91.7)		10 (83.3)	2 (16.7)	

## 4 Discussion

Pancreatic cancer is currently the fourth leading cause of cancer‐associated mortality in the Western world and is expected to surpass breast cancer, prostate cancer, and colorectal cancer by 2030 to become the second most common cause of cancer‐related death (Rahib *et al*., 2014). Despite extensive studies and exploration of the pathogenic and pathological mechanisms, pancreatic cancer is still refractory and recurrent to chemotherapy, and it remains a devastating malignant tumor characterized by a poor survival rate (Ryan *et al*., 2014). As a result, novel therapeutic strategies relying on the molecular biology of pancreatic cancer are urgently required. Here, we identified a paracrine OPN/integrin αvβ3/FOXM1 cascade that modulates the EMT and CSC‐like properties of pancreatic cancer, providing a promising therapeutic target for patients with pancreatic cancer.

Accumulating evidence has indicated that OPN promotes the progression of pancreatic cancer (Kolb *et al*., 2005; Lazar *et al*., 2010). However, the limitation of previous studies is that they usually focused on tumor cells and ignored the essential roles of stroma. Recently, the tumor microenvironment has attracted increasing attention. It is well established that tumorigenesis and progression are coevolutionary and reciprocal processes between tumor cells and the tumor microenvironment. PSCs, a major component of the tumor microenvironment of pancreatic cancer, are regarded as pivotal contributors to the development of pancreatic cancer. PSCs are relatively quiescent in normal pancreas, but they are activated in pancreatic cancer upon multiple stimulations, such as hypoxia, inflammation, and acidification (Bynigeri *et al*., 2017; Masamune and Shimosegawa, 2015). Activated PSCs form a paracrine niche for nearby PCCs, by which a large number of paracrine signaling pathways promote invasion, metastasis, and chemoresistance (Wang *et al*., 2017; Wu *et al*., 2017). The present study revealed that hypoxia induced PSC activation in a ROS‐dependent manner, which is consistent with our previous study (Lei *et al*., 2014). More importantly, we showed that hypoxia‐driven ROS‐induced PSC activation increased both the expression and the secretion of OPN. Notably, our results demonstrated that OPN was highly expressed in PSCs rather than PCCs, especially in activated PSCs driven by hypoxia, which emphasizes the critical roles of stroma in the biological effects induced by OPN.

Our further investigations showed that the expression of the β3 subunit of integrin had minimal differences in a panel of pancreatic cancer cell lines. However, we noted that the αv subunit of integrin, a key component of integrin αvβ3, was obviously differentially expressed in the above cell lines, which caused the significantly different expression of integrin αvβ3. Specifically, we observed that BxPC‐3 cells showed the highest expression, whereas CFPAC‐1 cells had little expression of the αv subunit of integrin. In addition, other cell lines showed moderate expression of the αv subunit of integrin, indicating that BxPC‐3 cells had the highest level of integrin αvβ3 expression while CFPAC‐1 cells had nearly no integrin αvβ3 expression. Our findings were quite similar to those of a previous study (Ji *et al*., 2012). Given that the differentially expressed patterns of potential receptors for OPN in a panel of pancreatic cancer cell lines, we chose BxPC‐3 and CFPAC‐1 cells for the following experiments. Our next investigations showed that activated PSCs driven by hypoxia promoted EMT and CSC‐like properties in BxPC‐3 cells via OPN in a paracrine manner by binding to its receptor, integrin αvβ3, as revealed by the elevated migratory, invasive, and sphere‐forming abilities, as well as the significant changes in EMT and CSC markers. However, the basic level of integrin αvβ3 was quite low in CFPAC‐1 cells. Therefore, OPN stimulation had little effect on EMT and CSC‐like properties in CFPAC‐1 cells. Our present study is the first to show that the PSC‐induced EMT and CSC‐like properties of PCCs are mediated by the paracrine OPN/integrin αvβ3 axis, which is a continuation and supplement of previous studies on OPN in pancreatic cancer. Interestingly, although integrin αvβ3 blockage using neutralizing antibody had little influence on EMT and CSC markers, it was observed that the migratory, invasive, and sphere‐forming abilities were attenuated in BxPC‐3 cells. These findings indicate that autocrine OPN signaling may also participate in the modulation of EMT and CSC‐like properties, similar to the paracrine regulation.

Dysregulated FOXM1 signaling has been identified in a wide range of cancers including pancreatic cancer (Pilarsky *et al*., 2004), and this dysregulation is considered as a key regulator of EMT and CSC‐like properties (Chiu *et al*., 2015). Several studies reported that overexpression of FOXM1 promoted EMT and endowed PCCs with self‐renewal and tumorigenic capacities (Bao *et al*., 2011; Li *et al*., 2014a). Therefore, we further investigated whether the downstream transcription factor FOXM1 was involved in the OPN/integrin αvβ3 axis‐induced EMT and CSC‐like properties. In line with the aforementioned findings, our results showed that FOXM1 was essential for the OPN‐induced EMT and CSC‐like properties in BxPC‐3 cells. Moreover, the activation of transcription factors is regulated by upstream signaling pathways, such as Akt and Erk, which are critical for the biological functions of FOXM1 in cancer progression (Yao *et al*., 2017). Our further investigations revealed that the OPN/integrin αvβ3 axis induced FOXM1 expression by activating the Akt and Erk signaling pathways. Taken together, our results indicate the complicated processes of the OPN/integrin αvβ3/FOXM1 cascade‐mediated EMT and CSC‐like properties in pancreatic cancer.

The lack of specific symptoms at the early stage and the paucity of biomarkers contribute to the poor diagnosis of pancreatic cancer. At the same time, since the tumor deteriorates easily and the metastasis rate is high, the prognosis of patients is still dismal (Zhou *et al*., 2017a). Thus, screening biomarkers for obtaining an early diagnosis and determining the clinical prognosis of pancreatic cancer are urgently needed. To support our *in vitro* observations, we retrospectively investigated the clinical value of OPN and FOXM1 expression in pancreatic cancer patients by IHC. Positive OPN staining was predominantly observed in stromal cells, while positive FOXM1 staining was mainly present in tumor cells. Moreover, OPN expression in stromal cells and FOXM1 expression in tumor cells were associated with poor clinical outcome. In addition, our further analysis showed that the expression levels of OPN and FOXM1 were significantly upregulated in pancreatic cancer tissues and were negatively correlated with patients’ OS and DFS according to the results from TCGA data, suggesting that these two genes might be considered as alternative diagnostic and prognostic biomarkers in pancreatic cancer.

**Figure 8:**
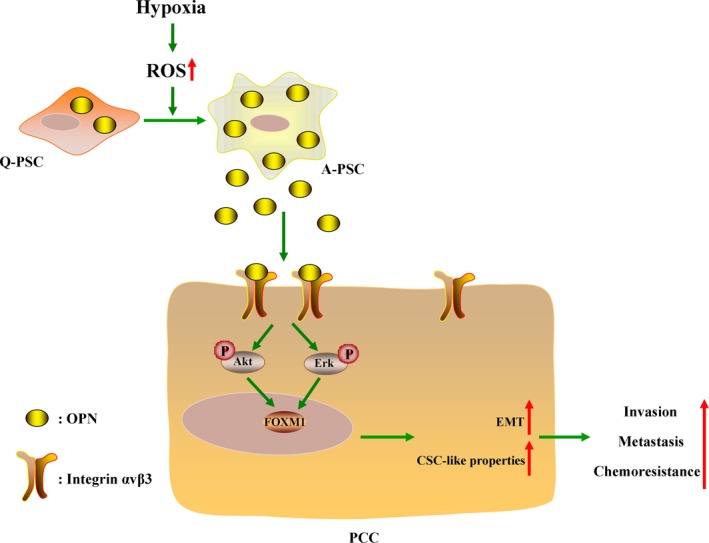
Schematic of the findings of the present study. The hypoxic tumor microenvironment promotes the activation of PSCs and increases the expression and secretion of OPN in PSCs in a ROS‐dependent manner. The OPN secreted by activated PSCs binds to integrin αvβ3 on the surface of PCCs, thus activating downstream signaling cascades, including the Akt and Erk pathways, which induces the expression of FOXM1. EMT and CSC‐like properties are then triggered, accelerating the progression of pancreatic cancer.

The proposed model (Fig. 8) presents a crosstalk between PCCs and PSCs in the hypoxic tumor microenvironment. Hypoxia aggravates the activation of PSCs and induces both the expression and the secretion of OPN in PSCs, by which paracrine OPN signaling promotes EMT and CSC‐like properties in PCCs through a FOXM1‐dependent manner.

## 5 Conclusions

In the present study, we provide evidence that hypoxic tumor microenvironment increases the expression and secretion of OPN in PSCs via a ROS‐dependent manner. More importantly, paracrine OPN signaling promotes EMT and CSC‐like properties in PCCs by activating the integrin αvβ3‐Akt/Erk‐FOXM1 cascade. In addition, our further data show that the expression levels of OPN and FOXM1 were significantly upregulated in pancreatic cancer tissues and were associated with poor clinical outcome. Our results provide novel insight into the mechanisms by which the tumor microenvironment promotes the progression of pancreatic cancer and potential targets that can be applied in the treatment of pancreatic cancer.

## Conflict of interest

The authors declare no conflict of interest.

## Author contributions

JC conceived the study, performed the experiments, and wrote the manuscript. JL and LS provided suggestions and participated in data analysis. TQ and YX contributed to the collection of the tissue specimens. KC and WQ contributed to clinical data analysis. WD, JL, and JM provided intellectual input and helped with the writing of the manuscript. QM and LH helped with the design of the study and the revision of the manuscript. All authors read and approved the final version of the manuscript for publication.

## Supporting information


**Table S1.** Primer sequences for quantitative real‐time RT‐PCR.

**Table S2.** The list of the utilized antibodies in the present study.
