# Supplementary material for: Hypoxia‐driven paracrine osteopontin/integrin αvβ3 signaling promotes pancreatic cancer cell epithelial–mesenchymal transition and cancer stem cell‐like properties by modulating forkhead box protein M1
Source: Mol Oncol. 2018 Dec 22;13(2):228–45. doi: 10.1002/1878-0261.12399 (PMC6360359; doi:10.1002/1878-0261.12399)
Supplement: Supplementary file 1 — Table S1. Primer sequences for quantitative real‐time RT‐PCR. [file MOL2-13-228-s001.docx]

**Table S1.** Primer sequences for quantitative real-time RT-PCR.

| **Genes** | **Primer Sequences** |
| --- | --- |
| OPN | Forward: 5′-ACCCTTCCAAGTAAGTCCAACG-3′ |
|  | Reverse: 5′-GGTGAGAATCATCAGTGTCATCTAC-3′ |
| FOXM1 | Forward: 5′-GGGCGCACGGCGGAAGATGAA-3′  Reverse: 5′-CCACTCTTCCAAGGGAGGGCTC-3′ |
| αv | Forward: 5′-TCCGATTCCAAACTGGGAGC-3′ |
|  | Reverse: 5′-AAGGCCACTGAAGATGGAGC-3′ |
| β3 | Forward: 5′-CTGGTGTTTACCACTGATGCCAAG-3′ |
|  | Reverse: 5′-TGTTGAGGCAGGTGGCATTGAAGG-3′ |
| Cyclin D1 | Forward: 5′-CCCTCGGTGTCCTACTTCAAATG-3′  Reverse: 5′-TCTGTTCCTCGCAGACCTCCA-3′ |
| MMP9 | Forward: 5′-CCGGACCAAGGATACAGTT-3′  Reverse: 5′-CGGCACTGAGGAATGATCTA-3′ |
| β-actin | Forward: 5′-AGGCTGTCCTGAATAAGCAG-3′ |
|  | Reverse: 5′-GGGCACGAAGGCTCATCATT-3′ |
