# Supplementary material for: Hypoxia‐driven paracrine osteopontin/integrin αvβ3 signaling promotes pancreatic cancer cell epithelial–mesenchymal transition and cancer stem cell‐like properties by modulating forkhead box protein M1
Source: Mol Oncol. 2018 Dec 22;13(2):228–45. doi: 10.1002/1878-0261.12399 (PMC6360359; doi:10.1002/1878-0261.12399)
Supplement: Supplementary file 2 — Table S2. The list of the utilized antibodies in the present study. [file MOL2-13-228-s002.docx]

**Table S2.** The list of the utilized antibodies in the present study.

| **Antibody** | **Dilution** | **Company** |
| --- | --- | --- |
| Mouse anti-α-SMA | 1:1,000(WB)  1:200(IF) | Boster |
| Mouse anti-CollagenⅠ | 1:1,000(WB) | Abcam |
| Rabbit anti-OPN | 1:1,000(WB)  1:150(IHC) | Abcam |
| Mouse anti-integrin αv | 1:1,000(WB) | Santa Cruz |
| Mouse anti-integrin β3 | 1:1,000(WB) | Santa Cruz |
| Rabbit anti-Sox2 | 1:1,000(WB) | Abcam |
| Rabbit anti-Oct4 | 1:1,000(WB) | Abcam |
| Rabbit anti-Nanog | 1:1,000(WB) | Abcam |
| Rabbit anti-E-cadherin | 1:1,000(WB) | CST |
| Rabbit anti-N-cadherin | 1:1,000(WB) | CST |
| Rabbit anti-Vimentin | 1:1,000(WB) | CST |
| Rabbit anti-Snail | 1:1,000(WB) | Abcam |
| Rabbit anti-FOXM1 | 1:1,000(WB)  1:150(IHC) | Abcam |
| Rabbit anti-Cyclin D1 | 1:1,000(WB) | CST |
| Rabbit anti-MMP9 | 1:1,000(WB) | CST |
| Rabbit anti-Akt | 1:1,000(WB) | CST |
| Rabbit anti-p-Akt (Ser473) | 1:1,000(WB) | CST |
| Rabbit anti-Erk1/2 | 1:1,000(WB) | CST |
| Rabbit anti-p-Erk1/2 (Thr202/Tyr204) | 1:1,000(WB) | CST |
| Mouse anti-β-actin | 1:10,000(WB) | Proteintech |
| Goat anti-rabbit IgG-HRP | 1:10,000(WB) | Abbkine |
| Goat anti-mouse IgG-HRP | 1:10,000(WB) | Abbkine |
| Goat anti-mouse dylight 488  (green) IgG antibody | 1:150 (IF) | Abbkine |
